# 78 kDa Glucose-Regulated Protein Attenuates Protein Aggregation and Monocyte Adhesion Induced by Angiotensin II in Vascular Cells

**DOI:** 10.3390/ijms21144980

**Published:** 2020-07-15

**Authors:** Stephanie Cicalese, Keisuke Okuno, Katherine J. Elliott, Tatsuo Kawai, Rosario Scalia, Victor Rizzo, Satoru Eguchi

**Affiliations:** Cardiovascular Research Center, Lewis Katz School of Medicine at Temple University, 3500 N. Broad Street, Philadelphia, PA 19140, USA; tug51315@temple.edu (S.C.); tun20059@temple.edu (K.O.); kelliott@temple.edu (K.J.E.); tuf88636@temple.edu (T.K.); rscalia@temple.edu (R.S.); rizzov@temple.edu (V.R.)

**Keywords:** protein aggregation, ER stress, inflammation, angiotensin II, vascular smooth muscle cells

## Abstract

Investigations of vascular smooth muscle cell (VSMC) phenotypic modulation due to angiotensin II (AngII) stimulation are important for understanding molecular mechanisms contributing to hypertension and associated vascular pathology. AngII induces endoplasmic reticulum (ER) stress in VSMCs, which has been implicated in hypertensive vascular remodeling. Under ER stress, 78 kDa glucose-regulated protein (GRP78) acts as an endogenous chaperone, as well as a master controller of unfolded protein response (UPR) to maintain protein quality control. However, the potential downstream consequences of ER stress induced by AngII on protein quality control and pro-inflammatory phenotype in VSMCs remain elusive. This study aims to identify protein aggregation as evidence of the disruption of protein quality control in VSMCs, and to test the hypothesis that preservation of proteostasis by overexpression of GRP78 can attenuate the AngII-induced pro-inflammatory phenotype in VSMCs. Increases in protein aggregation and enhanced UPR were observed in VSMCs exposed to AngII, which were mitigated by overexpression of GRP78. Moreover, GRP78 overexpression attenuated enhanced monocyte adhesion to VSMCs induced by AngII. Our results thus indicate that the prevention of protein aggregation can potentially mitigate an inflammatory phenotype in VSMCs, which may suggest an alternative therapy for the treatment of AngII-associated vascular disorders.

## 1. Introduction

Hypertension is a leading cause of mortality associated with cardiovascular diseases (CVD) [1]. Significant efforts have been made toward the prevention of and treatment for this disease so that the ever-expanding pandemic of CVD can be controlled. The renin angiotensin aldosterone system is an essential endocrine system that maintains blood pressure and homeostasis of sodium and water in mammalians [2]. However, overactivation of the renin angiotensin aldosterone system is believed to occur either systemically or locally, which significantly contributes to almost all kinds of CVD including hypertension and its complications [2,3]. Angiotensin II (AngII), a major bioactive peptide of the system, exerts its pathophysiological effects through the induction of vascular remodeling, amongst other maladaptive responses. In animal models of hypertension, AngII induces a hypertrophic, fibrotic and pro-inflammatory phenotype in vascular smooth muscle cells (VSMC) thus contributing to hypertensive vascular remodeling [4,5,6]. Using cultured VSMCs, several AngII signaling mechanisms that likely contribute to the hypertensive VSMC phenotype have been explored [6]. However, downstream cellular targets to mediate critical VSMC responses such as organelle dysfunction are still being investigated [7]. Prior studies demonstrated the involvement of endoplasmic reticulum (ER) stress in hypertensive vascular remodeling [8,9,10,11]. 

The ER is a highly dynamic organelle in which newly synthesized proteins are assembled and folded into their correct tertiary structures [12]. Accordingly, the ER is highly sensitive to alterations in its homeostasis. Upon accumulation of misfolded proteins, the unfolded protein response (UPR) is induced to preserve proteostasis through adaptations in protein folding, synthesis, and degradation. Specifically, the UPR promotes protein folding through the increased expression of molecular chaperone proteins, which include 78-kDa glucose-regulated protein (GRP78). Under non-stressed conditions, GRP78 binds to and inactivates UPR effectors such as inositol-requiring enzyme 1 α (IRE1α), protein kinase R-like ER kinase (PERK) and activating transcription factors (ATF) [13]. When misfolded proteins accumulate, however, GRP78 associates with them so that it detaches from and induces activation of the UPR effectors. Downstream mediators such as increased X-box-binding-protein 1 spliced isoform (XBP1s), ATF4, and cleaved ATF6 translocate to the nucleus following UPR activation to induce transcription of ER-associated degradation (ERAD) genes, autophagy machinery, and chaperones to restore ER protein folding competency [13]. However, chronic ER stress sustains UPR, which is associated with a heightened inflammatory response in multiple CVD models including those with AngII infusion [11,14,15,16]. Accordingly, interventions on ER proteostasis have been identified as promising therapeutics [17,18,19].

In animal models of neurodegenerative diseases such as Huntington’s and Alzheimer’s, pharmacological or genetic ER chaperoning appears effective to mitigate toxic aggregate accumulation in the brain [20,21]. Prior studies demonstrated the potential contribution of protein aggregates to inflammation and cardiovascular pathology [22,23]. The induction of pre-amyloid oligomers and protein aggregates have also been identified in human failing heart [24], as well as in the hearts of aged or AngII infused mice [25]. Since past studies have focused on aggregates in the heart and not the vasculature, the relationship between protein aggregation and the UPR system in VSMCs under hypertensive stimuli requires more understanding. Interestingly, resolving UPR activation under stress such as the IRE1α/XBP1s cascade prevented the development of atherosclerosis in mice [26,27]. Moreover, a prior study showed that cardiomyocyte-specific overexpression of GRP78 is protective against cardiac remodeling in a mouse model of ischemia/reperfusion [28]. Accordingly, we have tested the hypothesis that AngII induces protein aggregate accumulation in VSMCs, and that the preservation of proteostasis by adenoviral over-expression of GRP78 may mitigate a pro-inflammatory phenotype in VSMCs.

## 2. Results

### 2.1. Protein Aggregate Accumulation Is Induced by AngII 

**Figure 1:**
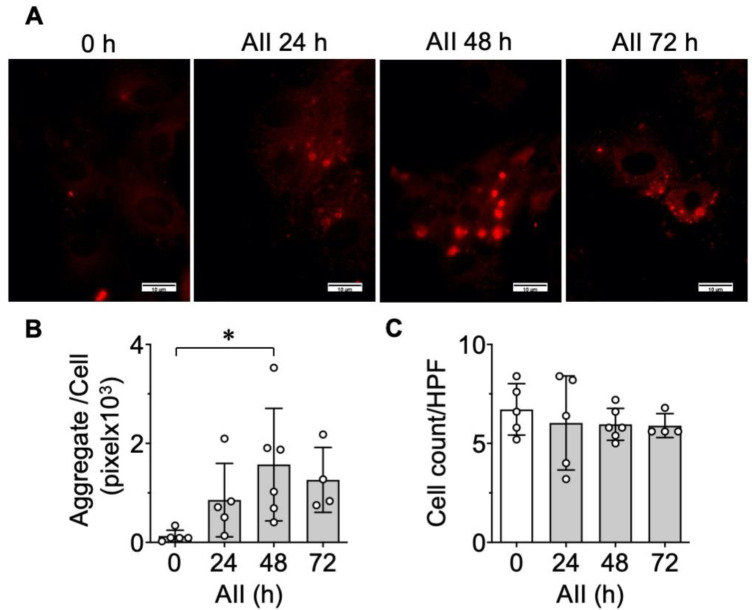
Induction of protein aggregation by angiotensin II (AngII) in vascular smooth muscle cell (VSMC). (**A**–**C**) The cultured rat aortic VSMCs were serum starved for 48 h. The VSMCs were stimulated with 100 nM AngII (AII) for 24–72 h as indicated. Representative Proteostat staining images are shown (**A**). The scale bar indicates 10 μm. The aggregate positive area per cell (**B**) and total attached cells (**C**) in high power field (HPF) were evaluated with ImageJ software. The bars in the graphs show the mean ± SD from three independent experiments with single or duplicated groups. * indicates *p* < 0.05.

It has been demonstrated that ER stress plays a critical role in the development of hypertensive vascular pathologies [8,9,11]; however, the cellular consequence of ER stress, such as aggregate formation, has not been explored in VSMCs. To evaluate protein aggregate accumulation, we used Proteostat dye to visualize aggresome formation [29]. AngII stimulation time-dependently increased aggresome formation assessed by the total aggregate area per cell, which was maximal at 48 h, whereas no alteration was observed in the total cell numbers (Figure 1).

### 2.2. GRP78 Chaperoning Reduces AngII-Induced Protein Aggregation and UPR in VSMCs

**Figure 2:**
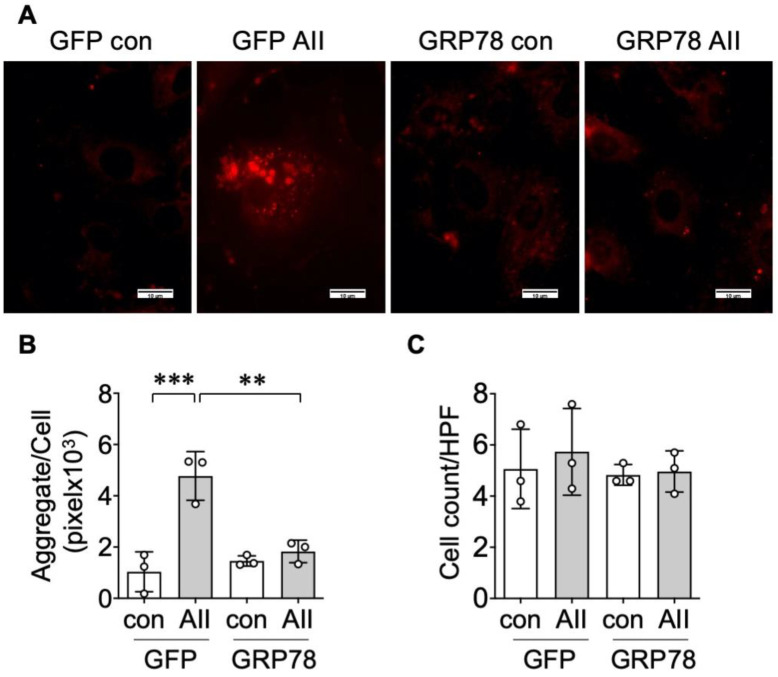
The transduction of 78 kDa glucose-regulated protein (GRP78) mitigates AngII-induced aggregate formation in VSMCs. (**A**–**C**) The rat aortic VSMCs infected with adenovirus encoding GRP78 or control green fluorescent protein (GFP) (100 multiplicity of infection (moi)) for 48 h were stimulated with 100 nM AngII (AII) for 48 h. Representative Proteostat staining images are shown. The scale bar indicates 10 μm. (**A**). The aggregate positive area per cell (**B**) and total attached cells (**C**) in HPF were evaluated with ImageJ software. The bars in the graphs show the mean ± SD from 3 independent experiments. ** indicates *p* < 0.01. *** indicates *p* < 0.001.

GRP78 has dual roles acting as an endogenous chaperone to interact with misfolded proteins that concurrently triggers UPR [13]. We observed that over-expression of GRP78 by adenovirus mitigated AngII-induced protein aggregate formation in VSMCs, whereas neither AngII nor GRP78 transduction altered cell numbers (Figure 2).

**Figure 3:**
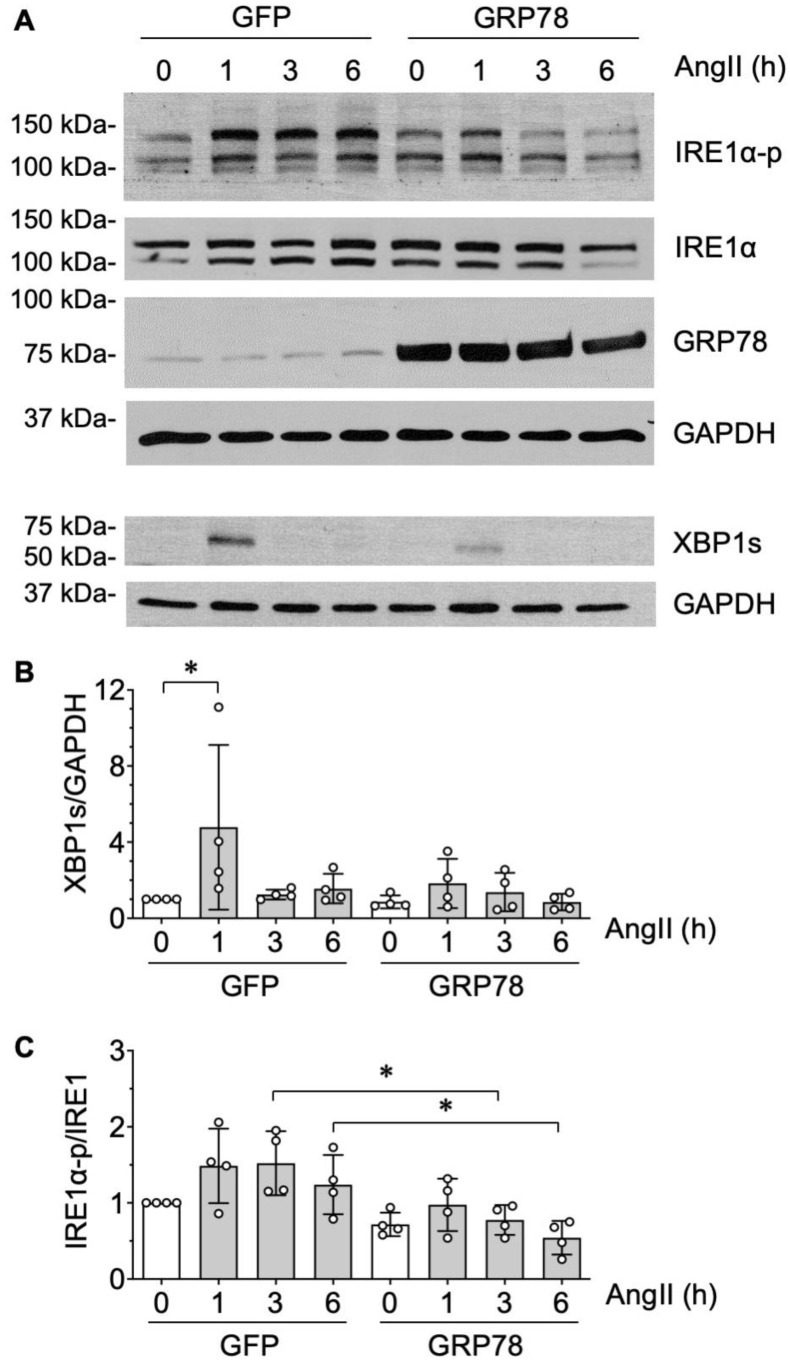
The inositol-requiring enzyme 1 α (IRE1α)/ X-box-binding-protein 1 spliced isoform (XBP1s) arm of unfolded protein response (UPR) is induced by angiotensin II in VSMCs. (**A**–**C**) The rat aortic VSMCs infected with adenovirus encoding GRP78 or control GFP (100 moi) for 48 h were stimulated with 100 nM AngII (AII) for 1–6 h and immunoblotting was performed as indicated. (**A**) Representative blots from 4 independent experiments. (**B**) Signal intensity was used to calculate the expression ratio of XBP1s to GAPDH. (**C**) Signal intensity was used to calculate the IRE1α Ser724 phosphorylation ratio to the total IRE1α. The bars in the graphs show the mean ± SD from 4 independent experiments. * indicates *p* < 0.05.

The IRE1α/XBP1s arm of ER stress signaling is the most deeply conserved UPR cascade. Upon ER stress triggering, dissociation between GRP78 and IRE1α, IRE1α dimerizes and transautophosphorylates, leading to the activation of its RNase activity. This, in turn, causes splicing of Xbp1 mRNA, subsequently generating XBP1s to initiate a UPR gene expression program to cope with ER stress [30]. In VSMCs, AngII induced a transient increase in XBP1s at 1 h, which was attenuated in VSMCs transduced with exogenous GRP78 (Figure 3A,B). A trend of increased IRE1α autophosphorylation was observed from 1 to 6 h time points assessed by immunoblotting with antibody against Ser724 phosphorylated IRE1α. A double banded pattern seen in both total and phosphorylated IRE1α may indicate ADP-ribosylation, since this post-translational modification causes an upward shift of IRE1α in SDS-PAGE [31]. A significant reduction in IRE1α phosphorylation was observed at 3 and 6 h time points in VSMCs transduced with exogenous GRP78 (Figure 3C). These data suggest that AngII stimulation causes protein misfolding and subsequent protein aggregation in VSMCs accompanied with induction of UPR. The induction of the IRE1α/XBP1s arm of UPR may be insufficient to prevent protein aggregation at the later 48 h time point, which can be mitigated by GRP78 over-expression. 

### 2.3. AngII-Induced Proinflammatory Phenotype Is Mitigated by GRP78 

**Figure 4:**
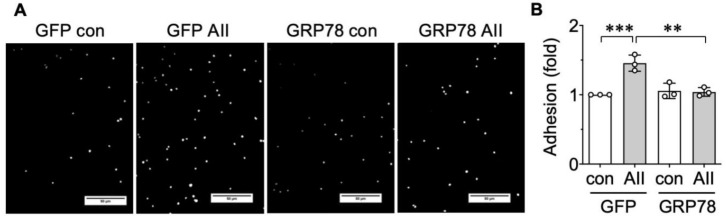
Upstream role of endoplasmic reticulum (ER) stress in VSMC inflammatory response induced by AngII. (**A** and **B**) The rat aortic VSMCs infected with adenovirus encoding GRP78 or control GFP (100 moi) for 48 h were stimulated with 100 nM AngII (AII) for 48 h. (**A**) Images of Hoechst labeled adherent THP-1 cells incubated on VSMC monolayer for 30 min. The scale bar indicates 50 μm. (**B**) The bars in the graphs show the mean ± SD from 3 independent experiments. ** indicates *p* < 0.01. *** indicates *p* < 0.001.

Prolonged UPR has been shown to be proinflammatory [32,33].Therefore, we next sought to determine if leukocyte adhesion to AngII exposed VSMCs could be mitigated by the ER chaperone treatment. THP-1 monocyte recruitment assay with VSMCs was utilized to simulate vascular inflammation [34]. The VSMCs stimulated for 48 h with AngII showed higher THP-1 adhesion compared with the baseline condition, which was significantly attenuated by GRP78 overexpression (Figure 4). Taken together, these data suggest that targeting proteostasis in VSMCs is an alternative strategy to alleviate vascular inflammation under the enhanced AngII activity in disease pathology, such as hypertension.

## 3. Discussion

**Figure 5:**
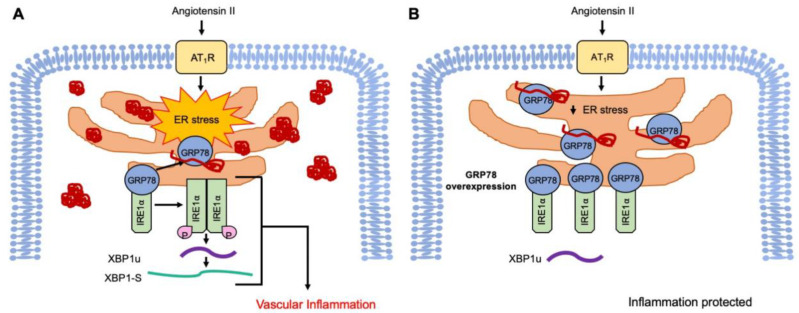
Proposed mechanisms for GRP78 mitigation of AngII-induced dysfunctional proteostasis and subsequent vascular inflammation. (**A**) Angiotensin II (AngII) via angiotensin II type 1 receptor (AT1R) induces ER stress. The proteostasis mechanism appears insufficient leading to protein aggregation in VSMC. GRP78 detaches from IRE1α to bind nascent misfolded peptide chains, and in turn leads to IRE1α dimerization and autophosphorylation. This leads to XBP1-S alternative splicing and potential UPRosome initiation, which activates VSMC proinflammatory phenotype, contributes to immune cell infiltration in the vasculature, and the overall progression of CVD. (**B**) The overexpression of GRP78 mitigates peptide misfolding into aggregates under AngII stimulation, while also curbing UPR signaling by maintaining its attachment to IRE1α and protecting monocyte adhesion/vascular inflammation.

The proteostasis network controls the health of the proteome by integrating pathways involved in protein synthesis, folding trafficking, and secretion and degradation, which declines with aging [35]. Although the contribution of protein aggregates to aging-related CVDs including hypertension has not been thoroughly investigated, a reduction in the buffering capacity of the proteostasis network in the vasculature may increase the risk of aortic stiffness and hypertension [8,36]. Specifically, our data presented here support the concept that the induction of ER stress and subsequent UPR by AngII potentially play a causative role in immune cell infiltration in the vasculature [7,37]. In pulmonary artery smooth muscle cells, endothelin-1 has been shown to stimulate inflammatory responses, including THP-1 adhesion via ER stress and UPR signaling [38]. In cultured endothelial cells (ECs), ER stress has been implicated in inflammation via vascular cellular adhesion molecule 1 induction [39]. In a concurrent study, we have also shown that treatment of a chemical chaperone, 4-phenylbutyrate, attenuated AngII-induced senescence as well as leukocyte adhesion in cultured EC [40]. Taken together, these data suggest that AngII induces protein aggregate formation and sustains UPR, which contributes to inflammation development, and thus sheds light on the potential anti-inflammatory ability of proteostasis mediation such as GRP78 transgene against vascular diseases (Figure 5).

A prior study characterized AngII-induced protein aggregates in the heart and cardiac myofibroblasts, and identified specific aggregated proteins using mass spectroscopy [25]. Based on proteomic profiling, AngII-induced hypertension seems to mimic some aspect of physiological aging because there was significant overlap in aggregated proteins purified from aged and hypertensive hearts [25]. In addition, previous research identified that vascular amyloidosis occurs in CVD, including the aggregation of transthyretin, apolipoprotein A-1, immunoglobin γ and medin [41]. It is therefore interesting to expand our research to test if these aggregates are formed in VSMCs upon AngII stimulation and contribute to the development of vascular pathology.

The present study demonstrated the activation of the IRE1α cascade of the UPR in response to AngII stimulation in VSMCs. In addition, to cope with ER stress by stimulating XBP1s-mediated UPR program, IRE1α forms a scaffold termed UPRosome to activate pro-inflammatory signaling responses including activation of c-Jun n-terminus kinase and nuclear-factor κB [30]. Accordingly, prior studies support the potential contribution of IRE1α and downstream inflammatory responses in metabolic syndrome and atherosclerosis [27,42]. Furthermore, XBP1s have been found to interact with the promoter region of proinflammatory cytokines such as interleukin-6 and tumor necrosis factor-α [43], which may also contribute to the ER stress regulation of THP-1 adhesion to VSMCs stimulated by AngII. However, further investigation into individual arms is necessary to understand the contribution of the UPR cascade in overall VSMC phenotype alterations.

In conclusion, the present study with forced GRP78 chaperoning represents protein aggregation as a novel target of VSMC dysfunction including inflammatory characteristics induced by AngII that may contribute to arterial pathology. Therefore, further understanding of the regulatory mechanisms of protein aggregation and the UPR system in vivo under hypertensive stimuli is of critical importance in order to explore therapeutic treatment options for CVD.

## 4. Materials and Methods

### 4.1. Culture of Rat Aortic VSMCs and Adenoviral Transduction

All animal procedures were performed with prior approval of the Temple University Institutional Animal Care and Use Committee (#4625, 21-Feb-2017) and in accordance with National Institutes of Health Guide for the Care and Use of Laboratory Animals. The thoracic aortic VSMCs were cultured from 12-week old male Sprague–Dawley rats (300–350 g Charles River Laboratories) by explant method as previously described [44,45]. To obtain VSMCs, rats were anesthetized with 5% isoflurane and euthanized by exsanguination and bilateral thoracotomy. VSMCs were pooled from 2–3 rats and renewed every 2 months. VSMCs were cultured in DMEM supplemented with 4.5 g/L D-glucose, 1 mM sodium pyruvate and 10% fetal bovine serum (FBS). VSMCs were used between passage 3–9 in all experiments. The adenoviral vectors encoding human GRP78 cDNA (32701, addgene, Watertown, MA, USA) and control green fluorescent protein (GFP) were constructed as previously described [46]. For gene transduction, 80–90% confluent VSMCs were serum-starved for 24 h and infected with 100 multiplicity of infection (moi) adenoviral GRP78 in serum-free DMEM containing 4 μg/mL polybrene for 90 min [47], followed by the addition of fresh serum-free media for another 24 h. The cells were then washed and incubated with serum-free media for 24 h and stimulated with AngII for the indicated time periods.

### 4.2. Proteostat Immunofluorescent Staining

A protein aggregation dye, ProteoStat Dual Detection Reagent (ENZO-51035-K100, Enzo Life Sciences, Farmingdale, NY, USA), was used to stain VSMCs seeded onto glass coverslips in 12 well dishes as described by the manufacturer. Proteostat is a molecular rotor that works in such a way as to spin with no fluorescence in the absence of aggregates; however, in their presence the dye slips into the exposed cavities of aggregated protein, thereby causing the dye rotation to be constrained, resulting in fluorescence [48]. Briefly, following respective treatments, VSMCs were washed twice with 1× phosphate buffered saline (PBS), then fixed with 4% formaldehyde for 30 min at room temperature. Next, the cells were washed three times with 1× PBS and then permeabilized with 0.5% Triton X-100 in 1× assay buffer with 3 mM EDTA, pH 8.0 for 30 min at 4 °C on a rocker. The cells were washed twice with PBS and then stained with Proteostat Dual Detection Reagent (diluted 1:2000, and Hoechst nuclear stain 1:1000, in 1× assay buffer). Following dye incubation, coverslips were washed with PBS and mounted onto glass slides, then imaged at 60× magnification. The aggregate size and number per 4′,6-diamidino-2-phenylindole (DAPI) count were counted for analysis in ImageJ 1.53e (NIH, Bethesda, MD, USA).

### 4.3. Immunoblotting

VSMCs were lysed in 1× SDS lysis, heated at 95 °C for 5 min, and subjected to SDS-PAGE gel electrophoresis as previously described [45,49]. Briefly, 10% acrylamide/bisacrylamide resolving gels were utilized and transferred onto 0.45 µm nitrocellulose membranes overnight at 30 V. The membranes were blocked for 1 h in 5% non-fat dry milk in TBS-T (0.01% Tween 20, VWRV0777, VWR, Radnor, PA, USA). Protein detection was performed using primary antibodies for GRP78 (Proteintech 11587-1-AP), phosphorylated IRE1α at Ser724 (NB100-2323, Novus, Centennial, CO, USA), IRE1α (NB100-2324, Novus, Centennial, CO, USA), XBP1s (D2C1F, Cell Signaling, Danvers, MA, USA), GAPDH (MAB374, MilliporeSigma, Burlington, MA, USA), incubated in TBS-T overnight at 4 °C, rocking. A secondary antibody incubation followed for 2 h at room temperature with subsequent chemiluminescence development on film for protein detection and quantification utilizing UN-SCAN-IT 6.2 software (Silk Scientific, Orem, UT, USA).

### 4.4. Monocyte Adhesion Assay

THP-1 monocyte adhesion assay was performed as previously described [34]. VSMCs were plated in 24 well dishes and serum starved for 48 h at 80–90% confluency. Media were replaced 1 h prior to AngII stimulation (100 nM) or pretreatments. Forty-eight hours after AngII treatment, the VSMC monolayer was washed 3 times with DMEM containing 0.2% bovine serum albumin (BSA). The THP-1 cells were cultured in RPMI 1640 media supplemented with 10 U/mL penicillin, 10 mg/mL streptomycin, β-mercaptoethanol and 10% FBS. The cells were maintained at 4–10 × 10^5^ cells/mL. THP-1 cells were collected for experiment at 30,000 cells/well and incubated with nuclear stain Hoescht (1:2000) in 0.2% BSA containing RPMI media for 30 min at 37 °C, 5% CO_2_. Following incubation, the THP-1 cells were washed with DMEM 0.2% BSA three times to remove excess dye with centrifugation to pellet cells in between. Subsequently, the THP-1 cells were resuspended and added to experimental VSMC conditions at a final concentration of 30,000 cells/well, and incubated on the VSMC monolayer at 37 °C, 5% CO_2_, for 30 min. Nonadherent THP-1 cells were gently washed with warmed 0.2% BSA media, and DAPI images were taken on a Olympus microscope at 20×. Five images per well were collected and analyzed for cell number to represent the number of adherent THP-1 cells per condition.

### 4.5. Statistical Analysis

Data are presented as mean ± SD (standard deviation). The comparisons were performed via one-way ANOVA with the post hoc Tukey method for multiple groups using Prism 8.4.2 software (GraphPad, San Diego, CA, USA). The differences were considered statistically significant at *p* < 0.05.

## Abbreviations

AngII	Angiotensin II
ATF	Activating transcription factor
BSA	Bovine serum albumin
CVD	Cardiovascular diseases
DAPI	4′,6-diamidino-2-phenylindole
DMEM	Dulbecco’s Modified Eagle’s Medium
DMSO	Dimethyl sulfoxide
ECs	Endothelial cells
ER	Endoplasmic reticulum
FBS	Fetal bovine serum
GFP	Green fluorescent protein
GRP78	78-kDa glucose-regulated protein
HPF	High power field
IRE1α	Inositol-requiring enzyme 1 α
moi	Multiplicity of infection
PBS	Phosphate buffered saline
PERK	Protein kinase R-like ER kinase
SD	Standard deviation
TBS	Tris buffered saline
UPR	Unfolded protein response
VSMCs	Vascular smooth muscle cells
XBP1s	X-box-binding-protein 1 spliced isoform
